# Fungal Genetics & Genomics: a call for manuscript submissions

**DOI:** 10.1093/g3journal/jkaa040

**Published:** 2021-02-06

The Genetics Society of America (GSA) journals *GENETICS* and *G3: Genes|Genomes|Genetics* have launched an ongoing series of publications on Fungal Genetics & Genomics.

The GSA and the fungal genetics community have a long relationship given their joint sponsorship of the Asilomar Fungal Genetics meeting, and *GENETICS* has a long and rich history of publishing landmark fungal genetics papers. Examples include: (1) landmark work done with *Neurospora crassa* as the model system by George Beadle and Art Tatum, whose studies were recognized via the Nobel Prize for their one gene–one enzyme hypothesis and discovery; (2) a series of publications by Jim Hicks, Jeff Strathern, and Ira Herskowitz and the independent pioneering contributions of Yasuji Oshima that provided robust genetic support for the cassette model of mating-type switching in *Saccharomyces cerevisiae*; and (3) additional landmark contributions highlighted below.

The Fungal Genetics & Genomics Series was launched across both GSA journals in February 2021 with an editorial and a block of research publications. It is overseen by Series Editors Leah Cowen (University of Toronto) and Joseph Heitman (Duke University). These exciting new resources aim to report and thereby further stimulate advances in genetics and genomics across a diversity of fungal species, and authors are invited to submit manuscripts to the series on an ongoing basis. Papers published in the series will continue to be highlighted across both *GENETICS* and *G3*.

Manuscripts will be reviewed and edited through the standard peer-review process and according to the usual high standards of the journals. Authors should submit to their journal of choice; some manuscripts submitted to *GENETICS* may be offered a transfer to *G3* as an option. To ensure proper routing of your submission, please choose the “Fungal Genetics & Genomics” article type in the submission systems and mention the series in your cover letter.

Conflicts of interest: None declared.
